# Effects of a 36-h Survival Training with Sleep Deprivation on Oxidative Stress and Muscle Damage Biomarkers in Young Healthy Men

**DOI:** 10.3390/ijerph15102066

**Published:** 2018-09-20

**Authors:** Ewa Jówko, Paweł Różański, Andrzej Tomczak

**Affiliations:** 1 Department of Physiology and Biochemistry, Faculty of Physical Education and Sport in Biała Podlaska, University of Physical Education in Warsaw, Akademicka 2, 21-500 Biała Podlaska, Poland; 2 Department of Uniformed Services and Combat Sports, University of Physical Education in Warsaw, 00-968 Warszawa, Poland; rozanski13@interia.pl (P.R.); atomczak33@wp.pl (A.T.)

**Keywords:** Keywords: lipid peroxidation, antioxidant capacity, blood prooxidant-antioxidant homeostasis, creatine kinase activity, students of physical education

## Abstract

The aim of this study was to analyze changes in oxidative stress and muscle damage markers during a 36-h survival training combined with sleep deprivation. The study included 23 male students of physical education (specialty: Physical Education for Uniformed Services), randomly divided into the survival or control group. The students in the survival group completed a 36-h survival training with moderate to low physical activity, without the possibility to sleep. The students in the control group performed only physical activity included in daily routines and had a normal sleep pattern. No significant changes in measured parameters were seen in the control group throughout the study period. In the survival group, plasma lipid hydroperoxides (LHs) and creatine kinase (CK) activity increased at 24 h and remained elevated up to 36 h (main effects for LHs: time, *p* = 0.006 and group × time, *p* = 0.00008; main effects for CK: time, *p* = 0.000001, group, *p* = 0.005, and group × time, *p* = 0.000001). A 12-h recovery was sufficient to normalize both LHs and CK to the pre-training level; in fact, the post-recovery LHs and CK levels were even lower than at baseline. Residual total antioxidant capacity (TAC) of plasma (without the major constituents: uric acid and albumin) was elevated at both 24 h and 36 h of survival training, but not following a 12-h recovery (main effects: group, *p* = 0.001 and group × time, *p* = 0.04). In turn, the activity of glutathione peroxidase (GPx) in whole blood and superoxide dismutase (SOD) in erythrocytes decreased between 24 h and 36 h of survival training (main group effect for GPx, *p* = 0.038 and SOD, *p* = 0.045). In conclusion, these findings imply that a 36-h survival training with sleep deprivation impairs enzymatic antioxidant defense, increases lipid peroxidation, and induces muscle damage. Our findings also indicate that at least in the case of young physically active men, a 12-h recovery after the 36-h period of physical activity with sleep deprivation may be sufficient for the normalization of oxidative and muscle damage markers and restoration of blood prooxidant-antioxidant homeostasis.

## 1. Introduction

Survival training belong to forms of physical activity that require a considerable amount of endurance and psychomotor preparation. It is based on long-lasting military training that often lasts over 24 h and combines ultra-prolonged physical effort with sleep deprivation [1,2].

It has been suggested that sleep deprivation may impair the ability to perform tasks that require additional energy expenditure [3]. Indeed, 30-h sleep deprivation and the associated muscle glycogen reduction and perceptual stress reduced sprint performance and slowed pacing strategies during an intermittent-sprint exercise in male team-sport athletes [4]. Moreover, a decrease in endurance performance during tasks with longer duration has been reported after sleep deprivation or recovery from thereof [5]. Moreover, in a previous study with participants in a survival camp, 36 h of sleep deprivation combined with 20 h of intermittent exercise resulted in a decrease in shooting performance and motor coordination [2]. In another experiment, participants exposed to 5-h intermittent moderate exercise during a 30-h period of sleep deprivation appeared to be more vulnerable in terms of negative mood disturbances and impaired reaction times to a greater degree than those experiencing sleep deprivation alone [6]. As pointed by the authors, the reduced capacity to respond quickly might be associated with a greater risk of accidents [6]. 

As known, among factors contributing to the deterioration of muscle function may be reactive oxygen species (ROS) [7]. It is generally accepted that acute strenuous exercise, but not exercise at low or moderate intensity [8], may promote the generation of ROS to a degree that cannot be counterbalanced by antioxidant defense [9]. ROS are highly reactive molecules that may be harmful to all cellular macromolecules, such as lipids, proteins, and DNA, and may cause, in turn, the release of cytosolic enzymes and other markers of cell damage [8]. Exercise-induced ROS may also exert detrimental effects on muscle function, impairing force generation and contributing to muscle fatigue [10] and a decrease in performance capacity [11]. 

Erythrocytes are susceptible to oxidative damage through continuous exposure to oxygen due to their high concentrations of polyunsaturated fatty acids and haem iron [12]. Thus, blood indices of oxidative stress may find application in sport practice as measures of inadequate recovery that may lead to overreaching [13]. 

To the best of our knowledge, none of the previous human studies analyzed the effects of survival training on oxidative stress markers, where the training was based on long-lasting exercise at low intensity in combination with sleep deprivation. Thus, the aim of the study was to analyze the effects of a 36-h survival training combined with sleep deprivation and a subsequent 12-h recovery period on selected blood parameters of oxidative stress and muscle damage biomarkers in young physically active men. As mentioned above, it is believed that exercise at low or moderate intensity does not induce oxidative stress but reinforces the enzymatic antioxidant system [8]. This, however, does not necessarily concern physical activity lasting over several hours. We hypothesized that the long-lasting exercise included in the survival training, even at low intensity, combined with sleep deprivation may disturb prooxidant-antioxidant homeostasis, and as a result induce oxidative stress and muscle damage. However, these parameters of oxidative stress and muscle damage are expected to improve after the 12-h recovery period, which includes overnight sleep. 

## 2. Material and Methods

### 2.1. Participants

The participants in our study were healthy male volunteers recruited among students of the University of Physical Education (specialty: Physical Education for Uniformed Services). Initially, 32 students volunteered to take part in the study. However, only 23 of them met the participation criteria prior to enrolment in the study. Exclusion criteria included use of tobacco products, alcohol consumption, history of a recent surgery or illness, and use of any medications or dietary supplements during 4 weeks prior to enrolment. All participants (*n* = 23) completed the study. None of the study participants practiced high-performance sports at the time of recruitment, and their organized physical activity was limited to the practical classes included in the study curriculum (8 h a week). Moreover, none of them had previous experience of sleep deprivation studies or reported abnormal sleep patterns prior to the study, as confirmed using the Pittsburg Sleep Quality Index (PSQI) to assess participants’ subjective sleep quality [14]. The total PSQI score of all participants was equal to or below 5, which indicates “good sleeper” according to Vardar et al. [14].

All participants provided their written informed consent to participate in the study. The protocol of the study was approved by the Local Ethics Committee at the Academy of Physical Education in Warsaw (Decision No.: SKE 01-16/2014).

### 2.2. Experimental Procedure

The students were randomly divided into two groups: survival (*n* = 12) and control (*n* = 11). The students in the survival group performed a 36-h survival training with sleep deprivation, followed by a 12-h recovery period. The students in the control group did not perform running or other rigorous physical activities apart from those related to their daily routines and had a normal sleep pattern (from 10.00 p.m. to 5.30 a.m.). 

The 36-h survival training was conducted in July at the Aquatic Sport Centre of the University of Physical Education in Rybitwy by Pisz. During the training, the subjects were constantly kept busy, involved in low physical activity and camp activities. The students needed to complete a 12-km march, row in a boat for 6 h, and paddle in a kayak for 4 h. Moreover, they were required to run, swim, climb, solve special tasks, and effectively navigate in a wild terrain, also overnight. The walk/run was held on a difficult, swampy terrain, and included additional special survival tasks. The overnight activities included crossing a lake by boat and an orienteering march on a diversified terrain under difficult conditions (night and day ambient temperature were 8 °C and 30 °C, respectively). The average heart rate (HR), that was monitored using the Sport-Tester Polar Team System (Polar Electro, Finland), ranged between 80 and 90 beats/min, representing 40–45% age–predicted HR_max_. However, individual HR values varied according to the type of physical activity, from 70–80 beats/min during the march, to 120–130 beats/min during rowing or kayaking. All activities were supervised by three licensed survival instructors, who also monitored sleep deprivation by paying close attention to each participant and preventing napping. The 36-h survival training was followed by a 12-h recovery period, which included activities related to daily routines and passive rest, with sleep time from 10.00 p.m. to 5.30 a.m. 

Throughout the experiment period, all participants received the same meals and had unlimited access to mineral water. The main meals were consumed at 7.00 a.m., 1.00 p.m., and 7.00 p.m. at the student canteen. The daily energy intake amounted to 3800 ± 120 kcal, with protein, fat, and carbohydrate contributing 14 ± 1, 30 ± 4, and 56 ± 3% of the dietary energy intake, respectively. 

### 2.3. Blood Sampling and Biochemical Analyses

Blood samples from the ulnar vein were collected at four time points: (1) prior to the survival training (at 6:00 a.m. on the first day, after an overnight fast; pre-training/baseline); (2) after 24 h of the survival training (at 6:00 a.m. on the second day, without an overnight rest; 24-h); (3) after 36 h of the training (at 6:00 p.m. on the second day, after a total of 36 h of sleep deprivation; 36-h); and (4) following a 12-h recovery (at 6:00 a.m. on the third day, after an overnight fast and sleep; 12-h rest). During the sampling at 24-h and 36-h, the subjects were at least 5–6 h after their last meal. In the control group, blood samples were collected at the same time points as in survival group.

Venous blood samples were drawn into heparinized test tubes and then centrifuged (for 10 min at 3000 × g at a temperature of 4 °C) to separate erythrocytes and plasma. Subsequently, the erythrocytes were washed three times with a cold isotonic saline solution. Both erythrocytes and plasma, as well whole blood, were frozen and stored at −80 °C until analysis.

Measured parameters were activity of superoxide dismutase (SOD) in erythrocytes, activity of glutathione peroxidase (GPx) in whole blood, total antioxidant capacity (TAC) of plasma, as well as the concentration of albumin (Alb), uric acid (UA), lipid hydroperoxides (LHs), and creatine kinase (CK) activity in plasma.

The SOD and GPx activities were determined with commercially available kits (RANSOD Cat. No. SD 125 and RANSEL Cat. No. RS 505, respectively; Randox, Crumlin, UK). The antioxidant enzyme activities were measured at 37 °C and expressed in U/g Hb. Hemoglobin was assessed using a standard cyanmethemoglobin method with a diagnostic kit (HG 1539; Randox, Crumlin, UK). The total antioxidant capacity of plasma (TAC) to scavenge ABTS radicals was measured using a chromogenic method with a commercially available kit (Cat. No. NX 2332, Randox, Crumlin, UK). The antioxidant capacity of samples was expressed as millimoles per liter of Trolox equivalents (6-hydroxy-2,5,7,8-tetramethylchroman-2-carboxylic acid). Plasma UA and Alb concentrations were determined with commercially available kits (Cat. No. K6580-200 and A6502-100, respectively; Alpha Diagnostics, Poland). Plasma LHs levels were determined as described previously [15]; the assay is based on the reaction of a chromogenic reagent, N-methyl-2-phenylindole, with malondialdehyde and 4-hydroxyalkenals at 45 °C. As a result, a stable chromophore is formed with maximum absorbance at 586 nm. Plasma CK activity was determined with the use of a diagnostic kit (Cat. No. C6512-100, Alpha Diagnostics, Poland).

### 2.4. Statistical Analysis

Statistical analysis was performed with Statistica version 12.0 software package (StatSoft, Krakow, Poland). Biochemical parameters in both groups were analyzed using a two-way ANOVA: 2 (groups: survival and control) × 4 (time points: pre-, 24-h, 36-h, 12-h rest), with main effects of time, group, and group × time interaction. The Bonferroni post-hoc test was used for multiple comparisons. The normal distribution of all variables was confirmed with the Shapiro–Wilk test and visual inspection (quantile distribution plots). Relationships within pairs of study variables were analyzed on the basis of Pearson’s coefficients of linear correlation. All values were reported as mean ± SD. The level of statistical significance was set at *p* < 0.05.

## 3. Results

**Figure 1:**
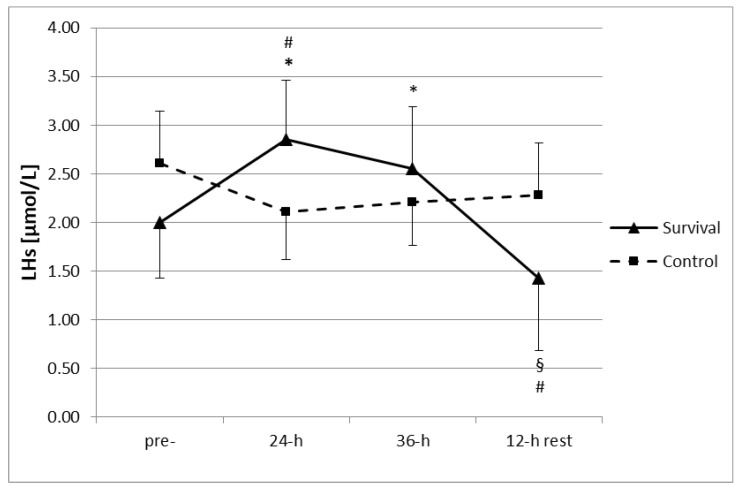
Changes in plasma concentration of lipid hydroperoxides (LHs) (µmol/L) in control group (*n* = 11) and survival group (*n* = 12) (i.e., 36-h survival training with subsequent 12-h rest period). Values are means ± SD. Main effects: time (*p* = 0.006); group × time (*p* = 0.00008); * significant difference (*p* < 0.05) as compared to pre-training (within same group); ^§^ significant difference (*p* < 0.05) as compared to pre-training, 24-h, and 36-h (within same group); ^#^ significant difference (*p* < 0.05) between survival and control groups (at same time point).

No significant differences were found between the two groups in regard to anthropometric characteristics (survival group: age 21 ± 0.7 years, body height 179.5 ± 5.6 cm, body mass 74.6 ± 8.0 kg; control group: age 21 ± 0.3 years, body height 180.5 ± 5.3 cm, body mass 80.1 ± 5.2 kg; *p* < 0.05). Moreover, no changes in measured parameters were observed in the control group throughout the study period. However, participation in the survival training resulted in an increase in plasma LHs (Figure 1). Following both 24 h and 36 h of training, plasma LHs were significantly higher than at baseline (*p* < 0.05). Moreover, the LHs levels at 24 h were also higher as compared to the control group. Subsequently, after a 12-h recovery, LHs were significantly lower than at pre-training levels, as well as at 24 h and 36 h of the training (*p* < 0.05). Plasma LHs after the 12-h recovery were below the respective level in the control group (*p* < 0.05).

**Figure 2:**
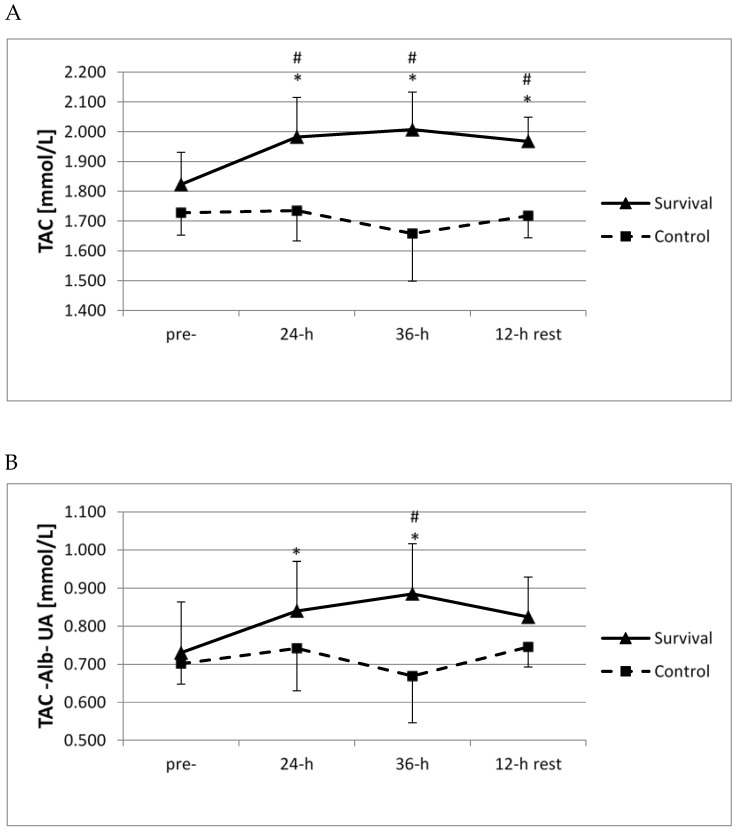
Changes in total antioxidant capacity (TAC) of plasma (**A**) and antioxidant capacity of plasma without albumin (Alb) or uric acid (UA) (**B**) in control group (*n* = 11) and survival group (*n* = 12). Values are means ± SD. Main effects for TAC: group (*p* = 0.00001); group × time (*p* = 0.005); Main effects for TAC-Alb-UA: group (*p* = 0.001); group × time (*p* = 0.04); * significant difference (*p* < 0.05) as compared to pre-training (within same group); ^#^ significant difference (*p* < 0.05) between survival and control groups (at same time point).

Both at 24 h and 36 h of survival training, as well as following the 12-h recovery, plasma TAC was significantly higher than at baseline and also in comparison with the control group (*p* < 0.05; Figure 2A). In both groups, no significant changes in plasma uric acid or albumin were documented at any of the analyzed time points (not shown). Moreover, a significant increase in residual TAC (without uric acid or albumin) was observed at both 24 and 36 h of survival training (*p* < 0.05; Figure 2B), and a difference between the two groups was seen at 36 h (*p* < 0.05; Figure 2B).

**Figure 3:**
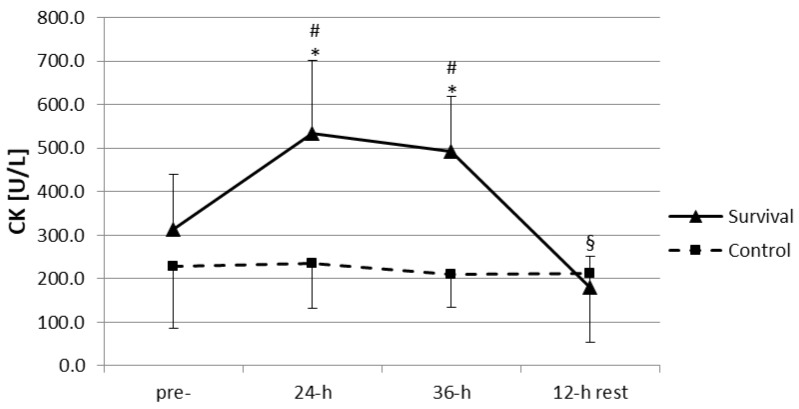
Changes in plasma activity of creatine kinase (CK) (U/L) in control group (*n* = 11) and survival group (*n* = 12). Values are means ± SD. Main effects: time (*p* = 0.000001); group (*p* = 0.005); group × time (*p* = 0.000001); * significant difference (*p* < 0.05) as compared to pre-training (within same group); ^§^ significant difference (*p* < 0.05) as compared to pre-training, 24-h, and 36-h (within same group); ^#^ significant difference (*p* < 0.05) between survival and control groups (at same time point).

The training-induced increase in plasma LHs was accompanied by an increase in plasma activity of CK (Figure 3). Plasma activity of CK at both 24 h and 36 h was significantly higher than at baseline and in the control group (*p* < 0.05). However, a 12-h recovery seemed to be sufficient for the normalization of muscle damage markers; plasma CK at this time point was not only significantly lower than at 24 h and 36 h of the training (*p* < 0.05), but also dropped off below its baseline level (*p* < 0.05). 

In the survival group, positive correlations were found between plasma activity of CK and plasma LHs at 36 h of the training and following the 12-h recovery (*r* = 0.51, *p* < 0.05 and *r* = 0.59, *p* < 0.05, respectively; not shown). A significant correlation between plasma CK and LHs was also observed when the results for all time points were pooled and analyzed together (*r* = 0.57, *p* < 0.0001; not shown). Using the same approach, we also found a weak albeit significant positive correlation between plasma activity of CK and plasma TAC (*r* = 0.30, *p* < 0.05; not shown).

**Figure 4:**
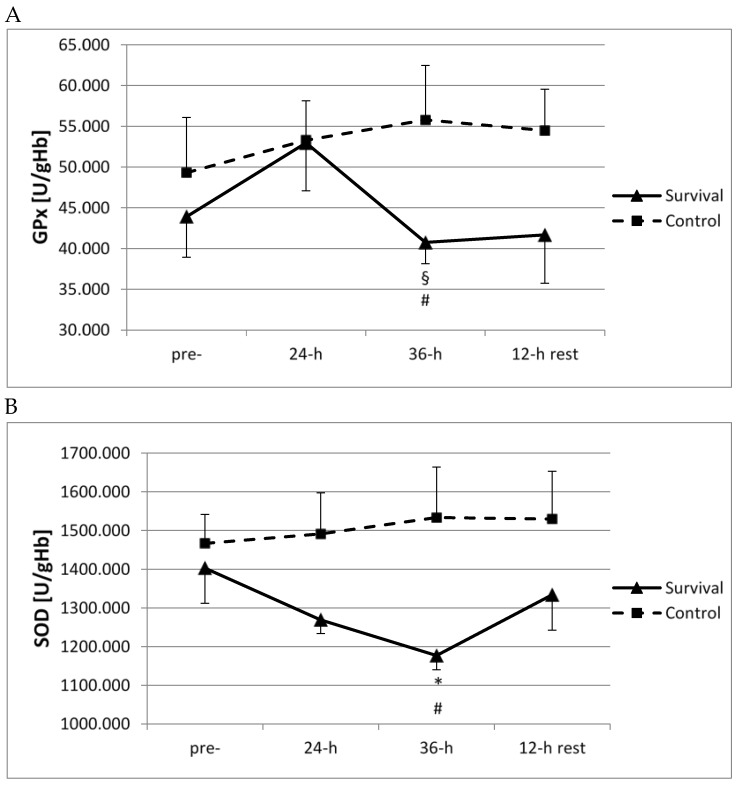
Changes in the activity of antioxidant enzymes in control group (*n* = 11) and survival group (*n* = 12). (**A**) Changes in whole blood activity of glutathione peroxidase (GPx) (U/gHb). Values are means ± SD. Main effects: group (*p* = 0.038); ^§^ significant difference (*p* < 0.05) as compared to 24-h (within same group); ^#^ significant difference (*p* < 0.05) between survival and control groups (at same time point); (**B**) Changes in erythrocyte activity of superoxide dismutase (SOD) (U/gHb). Values are means ± SD. Main effects: group (*p* = 0.045); * significant difference (*p* < 0.05) as compared to pre-training (within same group); ^#^ significant difference (*p* < 0.05) between survival and control groups (at same time point).

However, training-induced changes in the enzymatic antioxidant system followed the opposite pattern to that reported above. Whole blood activity of GPx at 36 h of survival training was significantly lower than at 24 h (*p* < 0.05; Figure 4A). Furthermore, SOD activity decreased at 36 h of survival training, as compared to pre-training activity (*p* < 0.05; Figure 4B). Moreover, for both GPx and SOD, significant differences were observed between the survival and control groups at this time point (*p* < 0.05; Figure 4A,B).

## 4. Discussion

This study demonstrated that even a short (36-h) survival training requiring low physical activity and imposing sleep deprivation may induce oxidative stress in young, healthy, and physically-active men. The presence of oxidative stress manifested as an increase in plasma lipid hydroperoxides, a marker of lipid peroxidation, observed already after 24 h of survival training. 

It is generally accepted that physical stress, such as that related to prolonged and chronic exercise, promotes the generation of reactive oxygen species, and erythrocytes are subjected to both mechanical and oxidative stress [10,16]. Reactive oxygen species are released into the circulation by immune, endothelial, and muscle cells, and interfere with the normal functioning of erythrocytes via peroxidation of lipid membranes [17]. 

The enhanced lipid peroxidation observed during the course of the 36-h survival training was accompanied by an increase in plasma TAC. As widely known, the major constituents of TAC are uric acid and albumin [18]. Moreover, physical exercise is postulated to induce catabolism of purine nucleotides to uric acid, which may be an explanation for exercise-induced changes in plasma TAC [19]. The exercise-induced increase in plasma albumin may also be a consequence of dehydration and hemoconcentration [20]. Therefore, we determined the concentrations of uric acid and albumin to exclude the potential contribution of these molecules to changes in plasma TAC. However, irrespective of the analyzed time point, we did not observe significant changes in plasma concentration of either albumin or hemoglobin (data not shown). This implies that no participants experienced dehydration, suggesting sufficient fluid intake. Moreover, plasma uric acid did not change throughout the experiment period. This is not surprising if we consider that our survival training included physical activities at relatively low intensity. It has been suggested that uric acid formation can arise from purine nucleotide degradation and fast-twitch fiber utilization during conditions of high energy utilization. Thus, exercise intensity rather than work output is the critical factor mediating increases in blood uric acid concentration [21]. According to the above, our results indicate that the increase in plasma TAC reflected changes in other molecules. Indeed, an increase in residual TAC (without uric acid or albumin) was observed at 24 h and 36 h of survival training. This phenomenon might result from the mobilization of low-molecular-weight antioxidants (such as reduced glutathione and vitamin C) from other tissues, and their release into circulation in order to counterbalance the enhanced synthesis of free radicals [22,23]. In line with these findings, an increase in TAC may be a simple marker of free radical overproduction [24]. However, the lack of measurement of nonenzymatic antioxidants such as reduced glutathione, vitamin C, or others in the present study may be considered a limitation and further research is needed to confirm this hypothesis.

Free radicals may mediate cell damage and vice versa: exercise-induced cell damage may contribute to enhanced synthesis of ROS. Exercise may promote microvascular dysfunction and cell damage via mechanical shear forces or through the disturbance of normal cellular metabolism. As a result, exercise triggers an inflammatory response, characterized by infiltration of affected areas by neutrophils and other phagocytic cells, followed by a respiratory burst involving the production of superoxide, hydrogen peroxide, and other ROS [25]. In our study, elevated levels of lipid peroxidation markers throughout the 36 h of survival training co-existed with an increase in plasma activity of CK. This implies that free radicals may play an important role in exercise-induced muscle injury, as suggested previously by Steinbacher and Eckl [10]. Another argument for the link between free radicals and muscle injury is the significant positive correlation of plasma CK activity with plasma LHs. Furthermore, we found a weak albeit significant positive correlation between plasma activity of CK and plasma TAC. These findings may suggest that free radicals contribute to muscle damage and stimulate the mobilization of low-molecular-weight antioxidants, probably as an adaptation mechanism activated in response to enhanced oxidative stress.

Unlike the non-enzymatic antioxidant potential of plasma, the enzymatic antioxidant system of our study participants seemed to be negatively affected by the 36-h survival training. Erythrocyte SOD and GPx are quite frequently used as markers of antioxidant status in human studies; however, the results are inconclusive, since exercise has been shown to cause an increase, decrease, or no alteration in the activity of these enzymes [16]. Our findings are consistent with the results of some previous studies that also documented an exercise-induced decrease in antioxidant enzymatic activity [26,27]. According to some authors, the loss of antioxidant activity after physical exercise may be explained by an exercise-induced oxidative damage to proteins that modify the catalytic activity of the enzymes [28,29]. Therefore, in our study, the impairment of the antioxidant system observed in the group of participants exposed to survival training might be associated with the partial inactivation of enzymatic proteins, resulting from their allosteric or covalent modifications induced by reactive oxygen species. The decrease in enzymatic antioxidant potential in our participants was due to the accumulation of ROS generated in response to exercise, as evidenced by the increase in plasma LHs as a marker of oxidative damage.

In our study, the main factor responsible for the above-mentioned shift in the prooxidative-antioxidative balance towards oxidative stress was probably an increase in oxygen consumption and aerobic metabolism to cover the increased energy expenditure during the physical activities involved in the 36-h survival training. We did not measure total energy expenditure, which is a limitation of this study. However, it should be emphasized that although our participants were exposed to prolonged exercise lasting 36 h, its intensity was relatively low. The results of several previous studies imply that the oxidation level during repeated, low-intense physical activity is either stable or decreased [30,31]. Therefore, the oxidative stress observed in our study participants was likely to be a consequence of exposure to not only physical but also psychological stressors related to, inter alia, sleep deprivation. To the best of our knowledge, none of the previous studies analyzed the combined effects of physical exercise and sleep deprivation on oxidative stress in humans.

Free radicals are postulated to accumulate during awakening, inter alia, due to enhanced metabolic activity, and are claimed to be responsible for the unfavorable effects of sleep deprivation [32,33]. During sleep deprivation, the ability to perform tasks that require additional energy expenditure may be impaired [3]. Moreover, evidence from some epidemiological and laboratory studies suggests that sleep deprivation may decrease tolerance to exercise in extreme weather, i.e., heat or cold [34]. The overnight activities included in the survival training used in this study included crossing a lake by boat and an orienteering march on a diversified terrain under difficult conditions, with ambient temperatures ranging between 8 °C during the night and 30 °C during the day. Therefore, despite the low intensity of the exercise, participants’ tolerance to it might be decreased by other factors, such as challenging conditions and sleep deprivation; these factors might also modulate the response of oxidative stress parameters to exercise. This may explain why peak levels of LHs and CK were observed at 24 h of survival training (i.e., after the overnight activities) and no further increase in these parameters was noted at 36 h. Moreover, in our study, the ability to differentiate forearm muscle strength deteriorated after the night activities (i.e., after 24 h of survival training), whereas a slight improvement in the results was observed in the further (daytime) training, after 36 h of survival training [35]. On the other hand, it is difficult to unambiguously determine to what extent physical activity affected the changes in biochemical parameters and to what extent the changes were due to sleep deprivation. Our study focused on the influence of the overall 36-h survival training on oxidative stress parameters, where such training involved both physical activities and sleep deprivation. That is why it would be impossible to analyze each of these components separately. Although our study did not consider the effect of the 36-h sleep deprivation itself on oxidative stress, another study with human participants indicated that even a lack of quality sleep may be associated with an increase in lipid peroxidation, a potential consequence of oxidative stress [36].

It has been postulated that sleep is a dynamic-resting state with antioxidative properties [37]. In line with the abovementioned findings, a 12-h recovery period, which included overnight sleep, turned out to be long enough to normalize lipid peroxidation and muscle damage markers in our participants, which even decreased below their pre-training levels. This decrease in oxidative damage parameters might result from the above-mentioned mobilization of low-molecular-weight antioxidants, as confirmed by an increase in residual TAC (without uric acid or albumin), which was observed at 24 h and 36 h of survival training. Furthermore, post-recovery activities of SOD and GPx did not differ significantly from their baseline values, despite the significant decrease in these parameters found at 36 h of the survival training. This may suggest restorative properties of sleep after long-term physical activity with sleep deprivation. 

Our results may have some practical implications. Aside from adventure races and survival, long-term physical activity with limited opportunity to sleep is also required from providers of various emergency services, namely firefighters, policemen, soldiers, crisis management specialists, and individuals who work in pro-active defense organizations and may become potential participants in joint actions with the military to combat natural disasters. Prolonged physical activity without an opportunity to sleep may contribute to unfavorable changes in prooxidant-antioxidant homeostasis and resultant tissue injury; one potential consequence of these changes is the deterioration of physical and mental performance. However, our results indicate that a 12-h recovery period may be sufficient to return blood prooxidant-antioxidant homeostasis. 

## 5. Conclusions

A 36-h survival training with sleep deprivation impairs enzymatic antioxidant defense, increases lipid peroxidation, and induces muscle damage. Our findings also indicate that at least in the case of young physically active men, a 12-h recovery after the 36-h period of physical activity with sleep deprivation may be sufficient for the normalization of oxidative and muscle damage markers and restoration of blood prooxidant-antioxidant homeostasis.
